# Mapping the Intratumoral and Peritumoral Microenvironment: Multilayered Shell ADC Analysis and Its Association with Multiparametric Biomarkers in Invasive Breast Cancer

**DOI:** 10.3390/tomography12040047

**Published:** 2026-03-31

**Authors:** Adil Aytaç, Bahar Yanık Keyik, Erdoğan Bülbül, Gülen Demirpolat, Gülay Turan

**Affiliations:** 1 Department of Radiology, Faculty of Medicine, Health Practice and Research Hospital, Balikesir University, 10145 Balikesir, Turkey; 2 Department of Pathology, Faculty of Medicine, Health Practice and Research Hospital, Balikesir University, 10145 Balikesir, Turkey

**Keywords:** breast neoplasms, magnetic resonance imaging, diffusion magnetic resonance imaging, tumor microenvironment, lymphatic metastasis

## Abstract

**Objective:** This study aimed to investigate the associations between intratumoral and peritumoral apparent diffusion coefficient (ADC) measurements and multiparametric biological markers in invasive breast cancer using a novel peritumoral analysis approach. **Materials and Methods:** In this retrospective study, 68 patients underwent 1.5 T breast magnetic resonance imaging. Following volumetric tumor segmentation, the peritumoral environment was analyzed using a segmentation-based, improved multilayered concentric shell model at distances of 0–2, 2–5, and 5–10 mm. The ADC values were normalized to contralateral parenchyma (rADC), and the intratumoral-to-peritumoral ADC ratios were calculated. Parameters were correlated with molecular subtypes, axillary metastasis, lymphovascular invasion (LVI), histologic grade, and Ki-67 index. **Results**: Lower intratumoral ADC and lower intratumoral-to-peritumoral ADC ratios were significantly associated with higher histologic grade, increased Ki-67, and axillary metastasis (*p* < 0.05). The 0–2 mm shell, representing the immediate invasion front, demonstrated the strongest associations with lymphovascular invasion and nodal involvement, while distance-dependent attenuation of effect sizes was observed across more distal peritumoral layers. **Conclusions:** The segmentation-based and improved multilayered shell model effectively captures the distance-dependent biological gradient of the peritumoral microenvironment. The intratumoral-to-peritumoral ADC ratios within the immediate 2 mm zone may provide complementary information regarding imaging markers of tumor aggressiveness when interpreted alongside absolute measurements. These findings suggest a potential role for these parameters as complementary imaging markers in preoperative risk stratification within a multiparametric framework.

## Simple Summary

This study investigates the prognostic significance of the peritumoral microenvironment in breast cancer. Using advanced MRI and a segmentation-based, improved multilayered shell model, we analyzed tissue at 0–2, 2–5, and 5–10 mm from the tumor margin. Our findings suggest that the diffusion characteristics within the immediate 2 mm “invasion front” may reflect important features of tumor aggressiveness and lymph node metastasis. By calculating the ratio between intratumoral and peritumoral diffusion measurements, we identified a potential complementary imaging marker. This method may contribute to preoperative risk assessment and support clinical decision-making, particularly when integrated with other clinical and imaging findings.

## 1. Introduction

Breast cancer is the most frequently diagnosed malignancy among women worldwide, accounting for approximately 2.3 million new cases annually and representing 11.7% of all cancers. Despite substantial advances in early detection and treatment, it continues to impose a considerable global burden, with approximately 685,000 deaths each year, remaining a leading cause of cancer-related mortality [1]. Accordingly, extensive efforts have focused on improving early detection in breast cancer through screening programs, advanced imaging techniques, and molecular-based approaches [2]. Nevertheless, pathological and molecular subtypes, genetic heterogeneity, and variations within the tumor microenvironment give rise to a broad phenotypic spectrum encompassing morphological characteristics, clinical presentation, and imaging findings [3].

The tumor microenvironment is a dynamic structure composed of stromal and immune cells, extracellular matrix components, and vascular elements, and tumor microenvironment interactions play a critical role in regulating tumor growth, invasion, metastasis, and response to therapy [4]. In invasive breast cancer, the peritumoral region extends beyond being a passive compartment surrounding the tumor; rather, it exhibits active biological alterations characterized by peritumoral lymphedema secondary to impaired lymphatic drainage, stromal cell activation, and structural and biochemical remodeling of the extracellular matrix [5]. This dynamic compartment constitutes a distinct microenvironment that interacts bidirectionally with tumor cells and is marked by a chronic inflammatory response resembling wound healing, along with pronounced increases in vascular density and permeability driven by enhanced angiogenesis. Collectively, these features shape tumor progression and invasive potential, positioning the peritumoral microenvironment as a critical biological determinant of disease prognosis [5].

In breast cancer, immunohistochemical markers—particularly estrogen and progesterone receptor expression, human epidermal growth factor receptor 2 (HER2) status, and the Ki-67 index, reflecting proliferative activity—play a central role in the comprehensive assessment of tumor biology [6]. Marker-based classification enables stratification of the disease into molecular subtypes, including luminal A, luminal B, HER2-enriched, and hormone receptor-negative subgroups, each exhibiting distinct biological behaviors in terms of proliferation rate, treatment responsiveness, and clinical course [7]. In addition, lymphovascular invasion, which denotes tumor cell infiltration into lymphatic and vascular structures, is regarded as a key histopathological indicator of aggressive tumor behavior and serves as a critical determinant in disease staging, prognostic assessment, and the selection of adjuvant treatment strategies due to its close association with axillary lymph node metastasis [8].

Breast magnetic resonance imaging (MRI) plays a pivotal role in the diagnostic evaluation of breast lesions owing to its high soft-tissue contrast and multiparametric imaging capability. In this context, diffusion-weighted imaging (DWI) provides complementary information for lesion characterization by offering qualitative and quantitative insights into tissue cellularity and microstructural organization, thereby contributing to improved diagnostic performance [9]. The fundamental physical principle of DWI is based on the quantitative assessment of the Brownian motion of water molecules within biological tissues through phase shifts in the magnetic resonance signal. Restriction of this motion by cellular density, cell membrane integrity, and extracellular matrix architecture enables DWI to indirectly yet biophysically meaningfully reflect the microstructural organization and biological behavior of tumor tissue [10]. Moreover, the apparent diffusion coefficient (ADC) derived from DWI quantitatively represents the degree of water diffusion within biological tissues and is considered more reliable for lesion characterization, as it provides standardized and reproducible measurements independent of signal intensity-based qualitative assessments [11].

In invasive breast cancer, intratumoral ADC values reflect diffusion characteristics associated with increased cellular density, reduced extracellular space, and preserved cell membrane integrity, and may therefore be biophysically linked to immunohistochemical markers of high proliferative activity, aggressive molecular subtypes, and hormone receptor-negative phenotypes [12]. In contrast, peritumoral ADC values are shaped by stromal remodeling, alterations in lymphatic and microvascular architecture, inflammatory processes, and disrupted interstitial fluid dynamics in the tumor surroundings. These microenvironmental changes may facilitate the invasion of lymphovascular structures and regional dissemination of tumor cells, thereby exhibiting closer associations with lymphovascular invasion and axillary lymph node metastasis [13]. Within this biophysical framework, the combined assessment of intratumoral and peritumoral ADC measurements, along with normalized parameters such as the intratumoral-to-peritumoral ADC ratio, which captures their relative difference, may enable an integrated characterization of cell-dominant tumor biology and microenvironment-dominant dissemination potential. This approach holds promise for quantitatively delineating biological aggressiveness and regional metastatic propensity in breast cancer [5,14]. In the existing literature, only a limited number of studies have explored the relationships between the biological behavior of breast cancer and histopathological features using peritumoral regions of interest, defined as narrow bands adjacent to the tumor margin, whole-tumor segmentation approaches, or multiple small region-of-interest (ROI) samplings placed around the tumor periphery [13,15,16]. However, the lack of standardization in ROI definition strategies for peritumoral ADC measurements remains a major methodological limitation, contributing to substantial heterogeneity across studies.

In this context, drawing inspiration from methodological approaches previously employed for peritumoral ADC assessment across different tumor types and studies, we synthesized and expanded existing measurement strategies within a broader framework to achieve a more consistent and comparable analysis [13,15,16]. Accordingly, we aimed to comprehensively evaluate intratumoral and peritumoral diffusion characteristics using a concentric peritumoral shell method defined by the outward, layered expansion of tumor segmentation. While previous studies have investigated the peritumoral environment using ROI-based measurements, most approaches treat the surrounding tissue as a homogeneous compartment. In contrast, the present study employs an automated multilayered 3D shell analysis to spatially map distance-dependent diffusion gradients within the peritumoral microenvironment. This strategy enables a more refined evaluation of spatial tumor–stroma interactions and may provide additional insight into biologically relevant stromal remodeling zones. Furthermore, intratumoral-to-peritumoral ADC ratios were calculated to characterize the relative diffusion relationship between tumor tissue and the surrounding microenvironment. In addition, normalized ADC (rADC) values were derived by referencing ADC measurements to corresponding anatomical locations within the contralateral breast parenchyma in order to reduce interindividual and parenchymal variability. Whereas prior studies have predominantly reported correlation analyses limited to single biological variables, such as lymphovascular invasion or molecular subtype [13,16], the present study adopts a multiparametric perspective. Specifically, we investigated the associations of intratumoral and peritumoral ADC-based quantitative parameters with immunohistochemical markers, molecular subgroups, histologic grade, lymphovascular invasion, and axillary lymph node metastasis, with the aim of more comprehensively elucidating the role of the peritumoral microenvironment in the biological aggressiveness and regional dissemination potential of breast cancer.

## 2. Materials and Methods

### 2.1. Study Design and Patient Selection

This study employed a retrospective, single-center, observational design. The study population was derived from patients evaluated at the Department of Radiology, Balıkesir University Faculty of Medicine Hospital, between 1 January 2020 and 1 September 2025. Patients with histopathologically confirmed invasive breast cancer who had undergone prediagnostic breast MRI were consecutively identified through a systematic review of institutional radiology and pathology databases.

This retrospective study was approved by the Balıkesir University Health Research Ethics Committee (decision number: 2025/7-13, approval date: 30 September 2025), and the study was conducted in accordance with the principles of the Declaration of Helsinki.

Inclusion Criteria:

Patients meeting all of the following criteria were included in the study:

- Histopathologically confirmed diagnosis of invasive breast cancer;
- Availability of prediagnostic breast MRI;
- Imaging studies of sufficient technical quality for quantitative analysis;
- Complete histopathological data, including the Ki-67 proliferation index, lymphovascular invasion status, and axillary lymph node metastasis status;
- Age ≥ 18 years.

Exclusion Criteria:

Patients meeting any of the following criteria were excluded:

- Diagnosis limited to in situ breast carcinoma;
- Availability of MRI examinations acquired after neoadjuvant or adjuvant therapy;
- Images unsuitable for quantitative ADC assessment due to motion artifacts, magnetic susceptibility effects, or other technical limitations;
- Incomplete histopathological data regarding immunohistochemical markers, molecular subtype, lymphovascular invasion, or axillary lymph node status;
- Tumors too small to allow for reliable ROI placement and ADC measurement;
- History of another primary malignancy or evidence that the breast lesion was of metastatic origin;
- Recent biopsy performed prior to MRI, with a potential risk of biopsy-related hemorrhage, edema, or altered diffusion characteristics.

### 2.2. Clinical and Pathological Data Collection

Clinical, histopathological, and immunohistochemical data were obtained through a comprehensive review of the hospital information management system and pathology archive records. The recorded clinical variables included patient age, tumor size, and axillary lymph node metastasis status. Pathological assessments were based on histopathological reports derived from surgical specimens or tru-cut biopsy samples. Tumor histologic type and histologic grade were documented according to the Nottingham modified Bloom–Richardson classification system.

The presence of lymphovascular invasion (LVI) was defined as the identification of tumor cells within endothelial-lined lymphatic or vascular spaces and was classified as positive or negative based on pathology reports. Axillary lymph node metastasis status was determined using the results of surgical axillary dissection or sentinel lymph node biopsy.

Immunohistochemical analyses included the evaluation of estrogen receptor (ER), progesterone receptor (PR), and human epidermal growth factor receptor 2 (HER2) status and the Ki-67 proliferation index. ER and PR expression were categorized as positive or negative according to the percentage of tumor cell nuclei exhibiting positive staining. HER2 status was assessed using standard immunohistochemical scoring criteria, with fluorescence in situ hybridization results considered for equivocal (2+) cases when applicable. The Ki-67 proliferation index was recorded as the percentage of positively stained tumor cell nuclei.

Based on these immunohistochemical parameters, tumors were classified into molecular subgroups, including luminal A, luminal B, HER2-enriched, and hormone receptor-negative (triple-negative) subtypes. All clinical, histopathological, and immunohistochemical variables were organized into a structured dataset to enable comparative analysis with imaging-derived parameters.

### 2.3. MRI Acquisition Protocol

All imaging was performed with a standardized breast MRI protocol on a 1.5 Tesla scanner (Ingenia, Philips Medical Systems, Best, The Netherlands) using a dedicated multichannel bilateral breast coil, with patients in the prone position.

The sequence protocols are detailed below:

- Axial T1-weighted turbo spin echo (T1W TSE): repetition time/echo time (TR/TE), 540/8.0 ms; field of view (FOV), 280 × 373 × 165 mm; slice thickness, 3 mm; matrix, 280 × 358 × 55.
- Axial DWI (single-shot echo-planar imaging): TR/TE, 11,532/86 ms;
  b-value, 1000 s/mm^2^; FOV, 280 × 368 × 165 mm; slice thickness, 3 mm; matrix, 112 × 148 × 55.
  ADC was generated automatically using vendor software (Philips Medical Systems, Best, The Netherlands).
- Axial VISTA-SPAIR: TR/TE, 2000/220 ms; FOV, 340 × 340 × 175 mm; slice thickness, 2 mm; matrix, 360 × 359 × 175.
- Axial three-dimensional T2-weighted fat-saturated imaging (3D T2 FS): TR/TE, 1300/130 ms.
  FOV, 360 × 360 × 175 mm; slice thickness, 2 mm; matrix, 360 × 359 × 175.
- Dynamic contrast-enhanced T1-weighted imaging (THRIVE): TR/TE, 5.1/2.5 ms; FOV, 300 × 372 × 175 mm; slice thickness, 2 mm; matrix, 300 × 371 × 175.

A gadolinium-based contrast agent was administered intravenously at a dose of 0.1 mmol/kg body weight for dynamic contrast-enhanced imaging. To enhance the assessment of contrast uptake, subtraction images were automatically generated using pre-contrast and post-contrast fat-suppressed T1-weighted dynamic series with a voxel-based subtraction technique provided by vendor software. Subtraction images were evaluated in conjunction with the dynamic contrast-enhanced sequences and other anatomical images.

### 2.4. Image Analysis and Measurement Methodology for Region of Interest (ROI)/Volume of Interest (VOI)

All image analyses were performed by a radiologist with extensive experience in MRI and 8 years of dedicated expertise in breast radiology. During image analysis, the interpreting radiologist was fully blinded to the histopathological results, immunohistochemical data, and clinical information. All imaging datasets were transferred to 3D Slicer (version 5.6.1; Brigham and Women’s Hospital, Boston, MA, USA), an open-source medical image analysis platform, and all segmentation and quantitative measurements were conducted within this environment.

Tumor segmentation was performed with reference to post-contrast fat-suppressed T1-weighted three-dimensional eTHRIVE sequences, on which lesion margins were most clearly delineated. When necessary, tumor boundaries were cross-referenced with fat-suppressed T2-weighted images to improve anatomical accuracy. DWI and ADC maps were spatially co-registered with anatomical sequences and incorporated into the analysis.

To assess intratumoral diffusion characteristics, a three-dimensional VOI encompassing the entire tumor volume was generated. VOI segmentation was performed across all slices using a semi-automated, manually refined approach to include only the solid tumor components. Areas of necrosis, cystic change, hemorrhage, and prominent vascular structures were carefully excluded. For each lesion, the mean intratumoral ADC (ADC_mean) value was calculated. This whole-tumor volumetric approach was selected to better capture tumor heterogeneity and to reduce sampling bias compared with single-slice ROI measurements.

To enable detailed evaluation of peritumoral microenvironmental diffusion characteristics, multilayered concentric peritumoral shell structures conforming to tumor geometry were generated based on the intratumoral VOI boundaries [17,18,19]. This approach allows for stepwise analysis of microenvironmental alterations as a function of increasing distance from the tumor margin. Accordingly, the peritumoral region was defined as three distinct volumetric layers extending outward from the tumor boundary:

- 0–2 mm peritumoral shell: Immediately adjacent to the tumor margin, representing the zone of highest invasive activity and stromal reaction;
- 2–5 mm peritumoral shell: An intermediate zone reflecting stromal remodeling and lymphatic and microvascular alterations;
- 5–10 mm peritumoral shell: A more distal region where tumor influence is relatively attenuated and transition toward surrounding breast parenchyma occurs.

**Figure 1:**
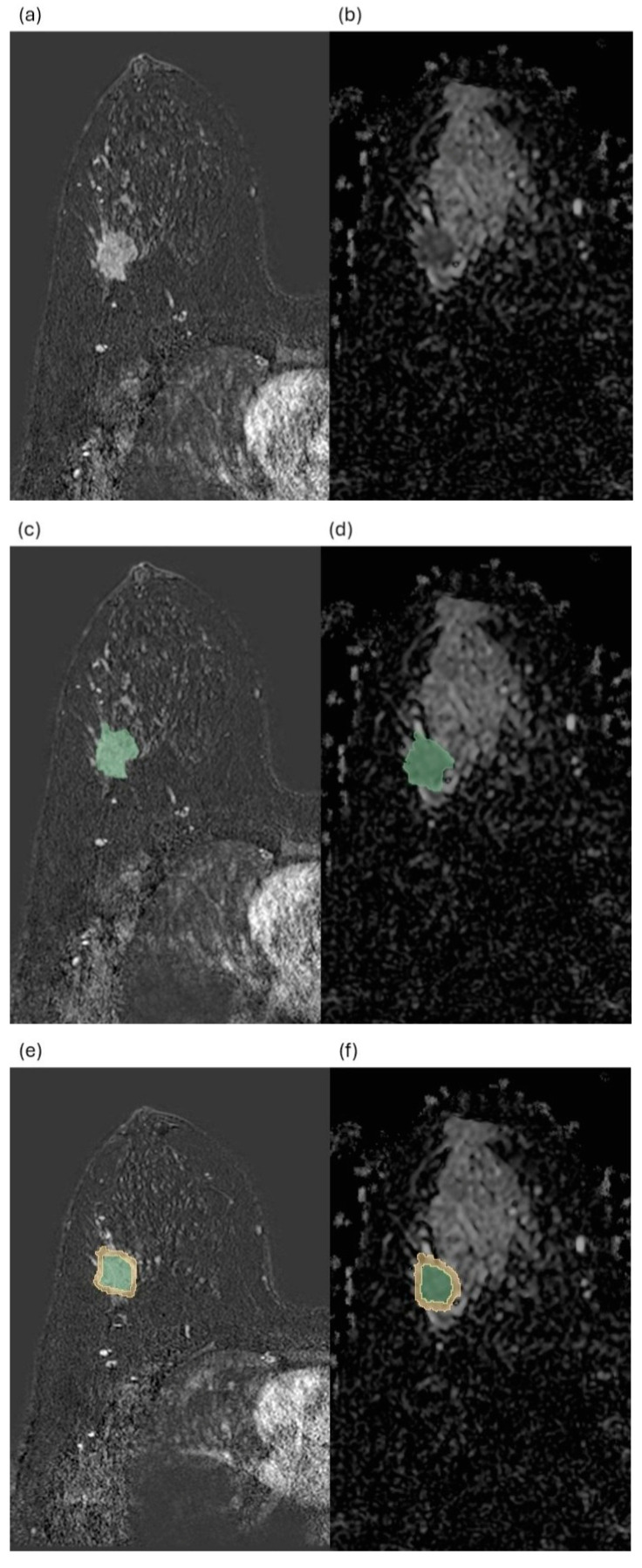

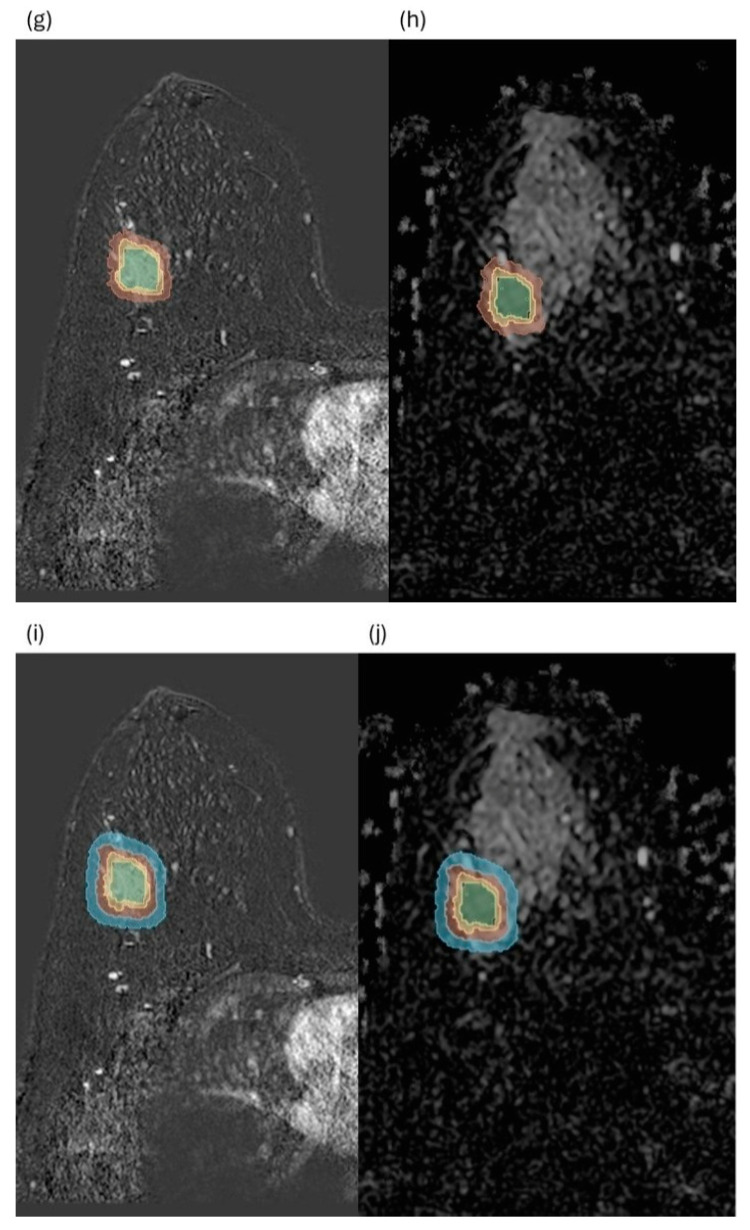
Representative example of intratumoral and peritumoral diffusion analysis using a segmentation-based multilayer concentric peritumoral shell model in invasive breast cancer. (**a**) Axial post-contrast T1-weighted image demonstrating the primary tumor. (**b**) Corresponding apparent diffusion coefficient (ADC) map. (**c**) Volumetric intratumoral segmentation overlaid on the post-contrast T1-weighted image (light green region). (**d**) Intratumoral region of interest overlaid on the ADC map (light green contour). (**e**) First peritumoral shell (0–2 mm) segmentation overlaid on the post-contrast T1-weighted image (yellow rim). (**f**) Corresponding ADC map showing the first peritumoral shell (0–2 mm). (**g**) Second peritumoral shell (2–5 mm) segmentation overlaid on the post-contrast T1-weighted image (orange rim). (**h**) Corresponding ADC map showing the second peritumoral shell (2–5 mm). (**i**) Third peritumoral shell (5–10 mm) segmentation overlaid on the post-contrast T1-weighted image (blue rim). (**j**) Corresponding ADC map showing the third peritumoral shell (5–10 mm).

Each peritumoral shell was generated by automatic outward expansion of the intratumoral VOI. Breast skin, pectoral muscle, and chest wall structures were excluded following automated processing, with additional manual corrections applied when necessary to ensure that the analysis was confined exclusively to breast parenchyma. During shell verification, care was taken to ensure that diffusion measurements remained confined to breast parenchymal tissue. Macroscopically evident adipose regions, large vessels, and other non-parenchymal structures were excluded when necessary through manual refinement of the semi-automated segmentation. In addition, ADC values within each shell were derived using voxel-wise averaging across the entire three-dimensional shell volume to minimize the influence of local signal heterogeneity and potential adipose tissue contamination. To ensure measurement precision within the narrow peritumoral regions (0–2 mm and 2–5 mm), several standardized procedures were implemented. All diffusion-weighted imaging datasets were first resampled to an isotropic voxel size of 1.0 × 1.0 × 1.0 mm to improve spatial consistency and reduce partial-volume effects. Resampling to isotropic voxels enabled more precise three-dimensional geometric expansion of the tumor boundary and improved the spatial definition of narrow peritumoral shells. Following semi-automated three-dimensional intratumoral segmentation, a one-voxel morphological erosion was applied to the tumor boundary to prevent intratumoral signal contamination into the peritumoral region. Subsequently, concentric peritumoral shells were automatically generated using a geometric expansion algorithm implemented in 3D Slicer. Final ADC values were calculated using voxel-wise averaging across the entire three-dimensional volume of each shell to obtain stable and representative quantitative measurements. Mean ADC (ADC_mean) values were calculated separately for each peritumoral shell. Representative examples of intratumoral and multilayered peritumoral shell-based image analyses are presented in Figure 1.

To reduce the interindividual variability in breast parenchymal characteristics and systemic diffusion differences, peritumoral ADC values were normalized using ADC measurements obtained from VOIs of equivalent size, defined at the same anatomical level within the contralateral breast. In addition, intratumoral-to-peritumoral ADC ratios were calculated to quantitatively express the relative relationship between intratumoral and peritumoral diffusion characteristics.

To assess measurement reproducibility, intratumoral and peritumoral ADC measurements were repeated by a different evaluator at a month interval in a randomly selected subset of cases. Interobserver agreement was subsequently evaluated using the intraclass correlation coefficient (ICC).

### 2.5. Statistical Analysis

The distributional properties of continuous variables were assessed using the Shapiro–Wilk test. Variables with a normal distribution are presented as the mean ± standard deviation, whereas non-normally distributed variables are expressed as the median (minimum–maximum). Categorical variables are summarized as frequencies and percentages (%). Comparisons between two groups for continuous variables were performed using the independent-samples *t* test or the Mann–Whitney *U* test, as appropriate based on data distribution. For comparisons involving three or more groups, one-way analysis of variance or the Kruskal–Wallis test was applied; when statistically significant differences were detected, appropriate post hoc analyses were conducted to identify the source of between-group differences.

Associations between categorical variables were evaluated using the chi-square test or Fisher’s exact test, as appropriate. Relationships between intratumoral and multilayered peritumoral ADC parameters and the Ki-67 proliferation index were examined using Pearson or Spearman correlation analyses, depending on data distribution. The predictive performance of ADC parameters for lymphovascular invasion and axillary lymph node metastasis was assessed using receiver operating characteristic (ROC) curve analysis; areas under the curve (AUCs) are reported with 95% confidence intervals, and optimal cut-off values were determined using the Youden index. The statistical significance of each AUC was assessed against the null hypothesis (AUC = 0.5) using the DeLong method. Pairwise comparisons between ROC curves were performed using the DeLong test. A *p*-value < 0.05 was considered statistically significant.

To enhance clinical interpretability, additional analyses were performed at clinically meaningful operating points reflecting potential diagnostic use scenarios. Specifically, sensitivity was evaluated at a fixed specificity level of 80%, corresponding to a rule-in strategy aimed at identifying patients with a higher likelihood of axillary lymph node metastasis while minimizing false positives. Conversely, specificity was evaluated at a fixed sensitivity level of 80%, reflecting a rule-out scenario where minimizing false negatives is prioritized. Furthermore, to assess incremental predictive value, additional multivariable logistic regression models were constructed, including tumor volume as a baseline variable followed by the addition of ADC-derived parameters. Model performance was compared using the area under the receiver operating characteristic curve (AUC).

To internally validate the predictive performance of the ADC-derived parameters for axillary lymph node metastasis, repeated 5-fold cross-validation with 200 repetitions and bootstrap resampling with 2000 iterations were performed. Repeated 5-fold cross-validation was preferred because of the relatively limited sample size and class distribution, enabling us to obtain more stable test set performance estimates. For each candidate parameter, the mean cross-validated area under the ROC curve (AUC) and its standard deviation were calculated. In addition, bootstrap-corrected AUC values and bootstrap-based 95% confidence intervals were obtained. Axillary lymph node status was treated as a binary outcome variable (pN− = 0, pN+ = 1).

Given the exploratory nature of this study and the biological interdependency of the evaluated ADC metrics, no formal adjustment for multiple comparisons was applied. Instead, internal validation using bootstrap resampling and repeated cross-validation was performed to evaluate the stability of the findings and to mitigate the potential risk of type I error.

ADC ratios reflecting the relative difference between intratumoral and peritumoral ADC values were included as independent variables in both group comparisons and predictive analyses. Intratumoral-to-peritumoral ADC ratios were calculated on a per-subject basis. Accordingly, the reported summary statistics represent the median of the 68 individual patient-level ratios rather than ratios derived from cohort median ADC values. This approach was adopted to better capture subject-level tumor–stroma interactions and may therefore yield values that differ from ratios calculated from aggregate medians. Measurement reproducibility was evaluated in a randomly selected subset of cases by repeating intratumoral and peritumoral ADC measurements at a monthly interval by a different observer, with interobserver agreement assessed using the ICC. A two-sided *p*-value < 0.05 was considered statistically significant for all analyses. Statistical analyses were performed using IBM SPSS Statistics for Windows, version 28.0 (IBM Corp., Armonk, NY, USA).

To enhance clinical interpretability, additional analyses were performed using a decision-oriented framework aligned with the intended clinical application. Specifically, the intratumoral/peritumoral ADC ratio (0–2 mm shell-1) was evaluated as a rule-in biomarker in the preoperative assessment of axillary lymph node metastasis, with an emphasis on specificity to minimize false positive classifications and potential overtreatment.

Accordingly, diagnostic performance was assessed at multiple clinically relevant operating points, including the Youden index-derived threshold, fixed specificity levels (approximately 80%, 85%, and 90%), and a fixed sensitivity level of approximately 80%.

To assess model stability and potential optimism, internal validation was performed using bootstrap resampling and repeated 5-fold cross-validation. Bootstrap analysis was also used to evaluate the stability of selected cut-off values by estimating their distribution across resampled datasets.

### 2.6. Temporally Independent Validation Cohort and Analysis

To further assess the generalizability of the proposed imaging parameters beyond the derivation dataset, a temporally independent validation cohort was constructed. This cohort consisted of 20 patients with histopathologically confirmed invasive breast cancer, retrospectively identified from our institutional database between 1 January 2018 and 31 December 2019. This cohort was fully independent from the primary study population (2020–2025) and was not involved in any stage of model development, parameter selection, or threshold determination, thereby minimizing the risk of data leakage. The same image processing pipeline used in the primary cohort was applied without modification. This included semi-automated volumetric tumor segmentation, automated generation of multilayered peritumoral shells using a geometric expansion algorithm, and calculation of ADC-derived parameters. Importantly, previously defined thresholds derived from the primary cohort (cut-off = 0.552 for the intratumoral/peritumoral ADC ratio, 0–2 mm shell) were applied directly to the validation dataset without re-optimization. No additional model fitting, parameter tuning, or threshold adjustment was performed in the validation cohort.

## 3. Results

### 3.1. Study Population

A total of 99 patients diagnosed with invasive breast cancer were initially identified. After applying the inclusion and exclusion criteria, 31 patients were excluded, and the final analysis was performed in 68 patients. The reasons for exclusion among the 31 patients were surgical treatment performed at an external institution (*n* = 19), MRI examinations not acquired at our institution (*n* = 7), incomplete histopathological data (*n* = 3), and insufficient image quality for quantitative assessment due to pronounced motion artifacts (*n* = 2).

### 3.2. Baseline Clinical and Pathological Characteristics

A total of 68 patients were included in the study. The patients’ ages ranged from 24 to 81 years, with a mean age of 48.5 ± 13.6 years and a median age of 45.5 years. Volumetric tumor segmentation performed using 3D Slicer (version 5.6.1) yielded a median tumor volume of 3999 mm^3^ (interquartile range [IQR]: 1123–9618 mm^3^).

On histopathological evaluation, invasive ductal carcinoma was the most frequent histologic subtype, accounting for more than two-thirds of cases (47/68, 69.1%). According to the Nottingham modified Bloom–Richardson grading system, intermediate-grade (grade 2) tumors were predominant (35/68, 51.5%), followed by low-grade (grade 1) tumors (22/68, 32.4%) and high-grade (grade 3) tumors (11/68, 16.2%). This distribution indicates a predominance of moderately differentiated tumors within the study population.

Immunohistochemical analysis demonstrated a high prevalence of hormone receptor positivity, with ER positivity observed in 83.8% (57/68) and PR positivity in 79.4% (54/68) of patients. HER2 positivity was identified in more than half of the cases (36/68, 52.9%). The Ki-67 proliferation index had a median value of 0.25 (IQR: 0.15–0.43).

Molecular subtype classification based on immunohistochemical findings revealed a marked predominance of the luminal B subtype (44/68, 64.7%), followed by luminal A (13/68, 19.1%), HER2-enriched (non-luminal) (6/68, 8.8%), and triple-negative breast cancer (5/68, 7.4%).

With respect to invasion-related features, lymphovascular invasion was present in one-quarter of cases (17/68, 25.0%), while perineural invasion was identified in approximately one-fifth of patients (14/68, 20.6%). Axillary lymph node metastasis was detected at diagnosis in 39.7% of patients (27/68), whereas no nodal metastasis was observed in the remaining cases (41/68, 60.3%). The demographic, histopathological, immunohistochemical, and molecular characteristics of the study population are detailed in Table 1.

### 3.3. Descriptive Statistics of ADC-Based Measurements

The intratumoral ADC values were found to be markedly lower than those of the peritumoral regions (intratumoral ADC: median: 0.88 × 10^−3^ mm^2^/s; IQR: 0.75–1.12 × 10^−3^ mm^2^/s). The peritumoral ADC values varied according to distance from the tumor margin, with lower values observed in the 2–5 mm peritumoral shell (shell-2) (median: 1.42 × 10^−3^ mm^2^/s; IQR: 1.27–1.56 × 10^−3^ mm^2^/s). In contrast, higher ADC values were detected in the 0–2 mm peritumoral shell (shell-1) (median: 1.52 × 10^−3^ mm^2^/s; IQR: 1.43–1.67 × 10^−3^ mm^2^/s) and the 5–10 mm peritumoral shell (shell-3) (median: 1.57 × 10^−3^ mm^2^/s; IQR: 1.47–1.77 × 10^−3^ mm^2^/s). The ADC values obtained from contralateral fibroglandular tissue used as reference were consistent with the peritumoral ADC range (median: 1.55 × 10^−3^ mm^2^/s; IQR: 1.50–1.69 × 10^−3^ mm^2^/s).

When normalized ADC parameters were evaluated, the normalized intratumoral ADC (intratumoral rADC) values were consistently well below unity (median: 0.57; IQR: 0.49–0.71). Normalized peritumoral ADC values showed limited variation across layers, with relatively lower values observed in the normalized shell-2 rADC (2–5 mm) (median: 0.91; IQR: 0.78–0.97), followed by the normalized shell-1 rADC (0–2 mm) (median: 0.97; IQR: 0.90–1.05) and the normalized shell-3 rADC (5–10 mm) (median: 0.99; IQR: 0.89–1.11).

The intratumoral-to-peritumoral ADC ratios were below unity across all peritumoral layers, supporting the predominance of diffusion restriction within the tumor relative to the surrounding microenvironment. Specifically, the intratumoral/peritumoral ADC ratio was 0.59 (IQR: 0.49–0.74) for the 0–2 mm shell, 0.66 (IQR: 0.55–0.83) for the 2–5 mm shell, and 0.55 (IQR: 0.48–0.73) for the 5–10 mm shell. Detailed descriptive statistics of ADC measurements are provided in Table 2.

### 3.4. Associations Between ADC-Based Parameters and Axillary Lymph Node Status

In cases with axillary lymph node metastasis (pN+), intratumoral ADC values were significantly lower than those observed in patients without nodal metastasis (pN0) (*p* = 0.0037). Similarly, normalized intratumoral ADC (intratumoral rADC) values were also significantly reduced in the pN+ group (*p* = 0.0145).

When ratio-based parameters were examined, the intratumoral-to-peritumoral ADC ratio for the 0–2 mm peritumoral shell (shell-1) was significantly lower in the pN+ group (*p* = 0.0066). The intratumoral-to-peritumoral ADC ratio for the 2–5 mm shell (shell-2) was lower in the pN+ group, but this difference did not reach statistical significance (*p* = 0.0802). In contrast, the ratio for the 5–10 mm shell (shell-3) was significantly lower in the pN+ group (*p* = 0.0089).

In contrast, the peritumoral ADC values for the 0–2 mm (shell-1), 2–5 mm (shell-2), and 5–10 mm (shell-3) layers, along with their corresponding normalized ADC parameters (shell-1 rADC, shell-2 rADC, and shell-3 rADC), generally exhibited higher values in the pN+ group; however, most of these differences did not reach statistical significance. Comparative results for the ADC, normalized ADC, and ratio-based metrics according to axillary lymph node status are summarized in Table 3.

In ROC analysis for predicting axillary lymph node metastasis, the intratumoral-to-peritumoral ADC ratio for the 0–2 mm peritumoral shell (shell-1) demonstrated the highest discriminative performance (AUC = 0.725; 95% CI: 0.590–0.841; *p* < 0.001). This was followed by intratumoral ADC (AUC = 0.710; 95% CI: 0.572–0.833; *p* = 0.002) and normalized intratumoral ADC (intratumoral rADC) (AUC = 0.677; 95% CI: 0.537–0.801; *p* = 0.011). Peritumoral ADC (0–2 mm shell-1) showed lower and non-significant discriminative performance (AUC = 0.634; 95% CI: 0.492–0.760; *p* = 0.068). In comparative model analysis, tumor size alone demonstrated an AUC of 0.655 (95% CI: 0.55–0.76; *p* = 0.015), whereas the combined model (tumor size + intratumoral/peritumoral ADC ratio) showed improved performance (AUC = 0.712; 95% CI: 0.61–0.81; *p* < 0.001). Statistical comparison of ROC curves using the DeLong test demonstrated a significant difference between the combined model and tumor size alone (*p* = 0.038).

**Figure 2:**
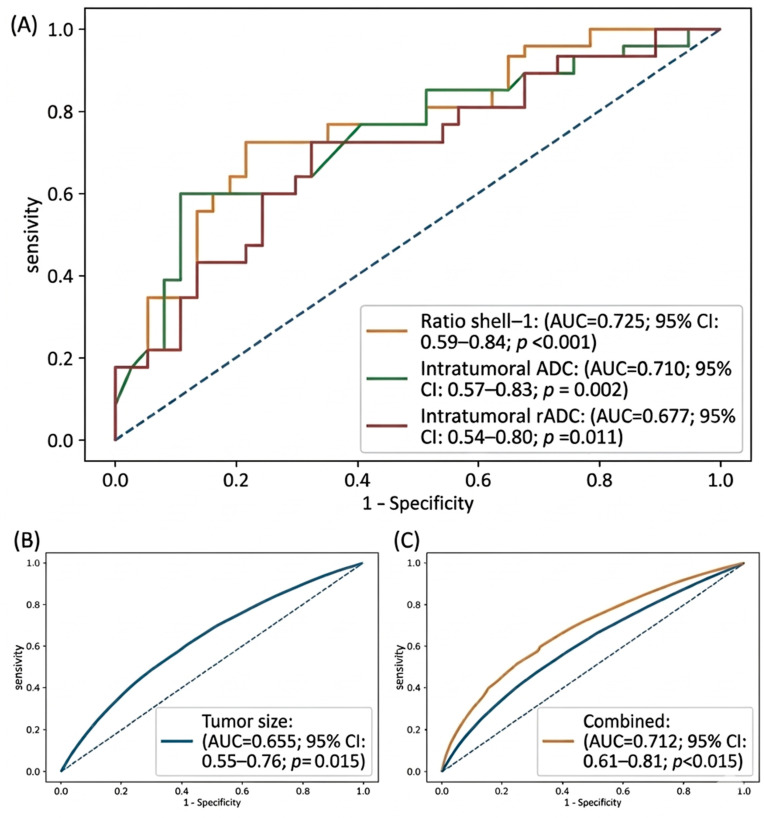
Receiver operating characteristic (ROC) curve analysis for predicting axillary lymph node metastasis (pN+). (**A**) Diagnostic performance of the top-performing ADC-derived metrics: The intratumoral/peritumoral ADC ratio (0–2 mm shell-1) achieved the highest discriminative power (AUC = 0.725; 95% CI: 0.59–0.84; *p* < 0.001), followed by intratumoral ADC (AUC = 0.710; 95% CI: 0.57–0.83; *p* = 0.002) and intratumoral rADC (AUC = 0.677; 95% CI: 0.54–0.80; *p* = 0.011). (**B**) ROC curve of conventional tumor size, showing moderate performance (AUC = 0.655; 95% CI: 0.55–0.76; *p* = 0.015). (**C**) Comparison between the combined model (tumor size + intratumoral/peritumoral ADC ratio) and tumor size alone. The combined model (AUC = 0.712; 95% CI: 0.61–0.81; *p* < 0.015) significantly outperformed tumor size alone (*p* = 0.038, DeLong test). The diagonal dashed line represents the reference for no discriminative ability (AUC = 0.5).

Based on optimal cut-off values determined using the Youden index, the intratumoral-to-peritumoral ADC ratio (0–2 mm shell-1) achieved moderate sensitivity and high specificity. Similarly, the intratumoral ADC and normalized intratumoral ADC (intratumoral rADC) provided moderate discriminative ability for axillary lymph node status. To further assess clinical applicability, at a fixed specificity level of 80%, the intratumoral/peritumoral ADC ratio (0–2 mm shell-1) demonstrated a sensitivity of 55.6%. Conversely, at a fixed sensitivity level of 80%, specificity was 36.6%. These findings suggest that this parameter may be more suitable for rule-in applications rather than for exclusion purposes. For clarity, detailed decision-oriented performance metrics are summarized in Table 4. Detailed performance metrics derived from ROC analysis are presented in Table 5, and ROC curves for the best-performing parameters are illustrated in Figure 2. Internal validation analyses showed that the predictive performance estimates were generally stable across resampling procedures. In repeated 5-fold cross-validation, the intratumoral-to-peritumoral ADC ratio for the 0–2 mm shell remained the best-performing parameter, with a mean AUC of 0.725 ± 0.131. The corresponding bootstrap-corrected AUC was 0.724 (95% CI: 0.590–0.841). Intratumoral ADC also demonstrated stable performance, with a cross-validated mean AUC of 0.709 ± 0.138 and a bootstrap-corrected AUC of 0.711 (95% CI: 0.569–0.838). Similarly, normalized intratumoral ADC showed a cross-validated mean AUC of 0.677 ± 0.140 and a bootstrap-corrected AUC of 0.675 (95% CI: 0.538–0.808). These findings indicate that the discriminatory performance of the main ADC-derived parameters was preserved after internal validation.

Threshold-dependent analyses demonstrated that the diagnostic performance of the intratumoral/peritumoral ADC ratio varied according to the selected operating point. While Table 4 presents performance at predefined clinical operating points, Table 6 provides a more detailed threshold-dependent analysis based on actual ROC-derived cut-off values. At the Youden index-derived cut-off (0.552), sensitivity and specificity were 66.7% and 78.0%, respectively. When specificity-oriented thresholds were applied, sensitivity decreased while specificity increased. At a specificity level of approximately 80%, sensitivity was 59.3%, whereas at higher specificity levels (approximately 85% and 90%), sensitivity further decreased to 51.9% and 33.3%, respectively. In contrast, at a fixed sensitivity level of approximately 80%, specificity was limited (36.6%), indicating reduced utility for rule-out purposes. Bootstrap-based internal validation demonstrated consistent model performance, with a bootstrap-corrected AUC of 0.702 compared to an apparent AUC of 0.715. Threshold stability analysis showed relatively narrow interquartile ranges across resampled datasets, supporting the internal robustness of the identified cut-offs (Table 6). These findings support the use of specificity-oriented thresholds for rule-in clinical applications.

In univariable logistic regression analysis, the intratumoral ADC (*p* = 0.0125), normalized intratumoral ADC (intratumoral rADC) (*p* = 0.0199), intratumoral-to-peritumoral ADC ratio for the 0–2 mm shell (shell-1) (*p* = 0.0019), and intratumoral-to-peritumoral ADC ratio for the 5–10 mm shell (shell-3) (*p* = 0.0174) were all significantly associated with axillary lymph node metastasis (pN+). In contrast, the normalized peritumoral ADC parameters and the intratumoral-to-peritumoral ADC ratio for the 2–5 mm shell (shell-2) did not demonstrate a statistically significant association (*p* = 0.0510).

In the multivariable logistic regression model adjusted for tumor volume, the intratumoral-to-peritumoral ADC ratio for the 0–2 mm shell (shell-1) remained significantly associated with pN+ (*p* = 0.0095), suggesting that this parameter may provide additional imaging-based information beyond tumor size. Tumor volume itself did not show a significant association with pN+ in the same model (*p* = 0.2183). In multivariable analysis, when added to tumor size, the intratumoral/peritumoral ADC ratio (0–2 mm shell-1) improved model performance (AUC: 0.655 vs. 0.712), indicating incremental predictive value beyond conventional predictors. Comparative multivariable model performance and incremental predictive value are summarized in Table 7. Detailed results of the logistic regression analyses are presented in Table 8.

### 3.5. Temporally Independent Validation Analysis

A temporally independent validation cohort consisting of 20 patients was analyzed to assess the reproducibility of the primary findings. In this cohort, the intratumoral/peritumoral ADC ratio (0–2 mm shell-1) demonstrated an area under the receiver operating characteristic (ROC) curve (AUC) of 0.75 (*p* = 0.046). Using the predefined cut-off value of 0.552, sensitivity was 50.0% and specificity was 80.0%. These findings are comparable to those observed in the primary cohort (AUC: 0.725, *p* < 0.001; specificity approximately 78%), indicating preservation of discriminative performance. Importantly, the direction of association was consistent with the primary analysis, with lower intratumoral/peritumoral ADC ratios associated with axillary lymph node metastasis. Although sensitivity was slightly reduced, specificity remained stable at the predefined rule-in operating point. A detailed comparison of baseline characteristics and diagnostic performance between the temporally independent validation cohort and the primary cohort is presented in Table 9. These results support the robustness and reproducibility of the proposed imaging parameter in a temporally independent setting.

### 3.6. Associations Between ADC-Based Parameters and Lymphovascular Invasion

In cases with lymphovascular invasion (LVI+), both intratumoral ADC and intratumoral rADC values were significantly lower than those observed in the LVI− group (*p* = 0.022 and *p* = 0.041, respectively). Similarly, the intratumoral-to-peritumoral ADC ratio for the 0–2 mm peritumoral shell (shell-1) was significantly reduced in LVI+ cases (*p* = 0.022).

In contrast, no statistically significant differences were observed between the LVI-positive and LVI-negative groups with respect to peritumoral ADC values across the 0–2 mm (shell-1), 2–5 mm (shell-2), and 5–10 mm (shell-3) layers, or for normalized peritumoral ADC values (shell rADC) (all *p* > 0.05). Although the intratumoral-to-peritumoral ADC ratios for the 2–5 mm (shell-2) and 5–10 mm (shell-3) layers tended to be lower in the LVI+ group, these differences did not reach statistical significance (*p* = 0.100 and *p* = 0.060, respectively). Comparative results of ADC metrics according to lymphovascular invasion status are summarized in Table 10.

### 3.7. Differences in ADC-Based Parameters Across Molecular Subtypes

In the Kruskal–Wallis analysis comparing molecular subtypes, intratumoral ADC (*p* < 0.001) and normalized intratumoral ADC (intratumoral rADC) (*p* = 0.001) demonstrated statistically significant differences across subgroups. In post hoc pairwise comparisons, the intratumoral ADC values in the luminal A group were significantly higher than those in the luminal B (combined HER2-negative and HER2-positive subtypes) (Bonferroni-corrected *p* = 0.017) and HER2-enriched (*p* = 0.001) groups. No significant differences in intratumoral ADC were observed between triple-negative breast cancer (TNBC) and the other molecular subtypes (all adjusted *p* > 0.05).

Similarly, the normalized intratumoral ADC values differed significantly among molecular subtypes, with post hoc analysis revealing higher values in the luminal A group compared with the luminal B (combined) (*p* = 0.024) and HER2-enriched (*p* = 0.002) groups.

Among ratio-based metrics, the intratumoral-to-peritumoral ADC ratio for the 0–2 mm shell (shell-1) (*p* = 0.005) and the 5–10 mm shell (shell-3) (*p* = 0.008) showed significant differences across molecular subtypes. Post hoc analysis demonstrated that the luminal A group had higher intratumoral-to-peritumoral ADC ratios for the 0–2 mm shell compared with the HER2-enriched group (*p* = 0.003). For the 5–10 mm shell, the luminal A values were significantly higher than those observed in both the luminal B (combined) (*p* = 0.031) and HER2-enriched (*p* = 0.004) groups.

**Figure 3:**
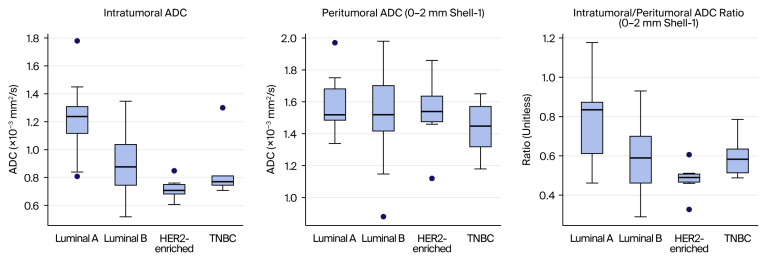
Box-and-whisker plots showing the distribution of intratumoral ADC, peritumoral ADC (0–2 mm shell-1), and intratumoral/peritumoral ADC ratio (0–2 mm shell-1) across molecular subtypes (Luminal A, Luminal B [combined], HER2-enriched, and triple-negative breast cancer). Boxes represent the interquartile range, the horizontal line indicates the median, whiskers denote the range excluding outliers, and dots represent outlier values.

Although the overall comparison for the intratumoral-to-peritumoral ADC ratio of the 2–5 mm shell (shell-2) reached statistical significance (*p* = 0.034), pairwise group comparisons did not retain significance after correction for multiple testing (all adjusted *p* > 0.05). In contrast, the peritumoral ADC values across all shells (0–2, 2–5, and 5–10 mm) and their corresponding normalized peritumoral ADC parameters (shell-1/2/3 rADC) did not differ significantly among molecular subtypes (all *p* > 0.05). Comparative results of ADC and rADC metrics according to molecular subtype are presented in Table 11, and the distributions of the key parameters across molecular subtypes are illustrated in Figure 3. Due to the relatively small sample sizes in the HER2-enriched and triple-negative subgroups, these results should be interpreted with caution, as the limited number of cases may reduce statistical power and increase the risk of unstable estimates.

### 3.8. Associations of ADC-Based Parameters with Histological Grade and Ki-67 Proliferation Index

Associations between histologic grade and ADC-based diffusion parameters were evaluated using Spearman’s rank correlation analysis (Table 8). A moderate, statistically significant inverse correlation was observed between intratumoral ADC values and histologic grade (ρ = −0.456, *p* < 0.001), indicating progressively greater intratumoral diffusion restriction with increasing histologic grade. Similarly, normalized intratumoral ADC (intratumoral rADC) demonstrated a significant negative correlation with histologic grade (ρ = −0.312, *p* = 0.010).

Among ratio-based parameters, the intratumoral-to-peritumoral ADC ratio for the 0–2 mm shell (shell-1) showed a significant inverse correlation with histologic grade (ρ = −0.346, *p* = 0.004). Weaker yet statistically significant negative correlations were also observed for the 2–5 mm shell (shell-2) (ρ = −0.291, *p* = 0.017) and the 5–10 mm shell (shell-3) (ρ = −0.268, *p* = 0.028). In contrast, no significant correlations were identified between peritumoral ADC values and histologic grade across any shell (all *p* > 0.05).

The relationships between ADC-based parameters and the Ki-67 proliferation index were also assessed using Spearman correlation analysis (Table 12). Intratumoral ADC exhibited a moderate, statistically significant inverse correlation with Ki-67 (ρ = −0.463, *p* < 0.001), suggesting increasing diffusion restriction with higher proliferative activity. Likewise, normalized intratumoral ADC was negatively correlated with Ki-67 (ρ = −0.337, *p* = 0.006).

Regarding ratio-based metrics, the intratumoral-to-peritumoral ADC ratio for the 0–2 mm shell (shell-1) demonstrated a strong and significant inverse correlation with Ki-67 (ρ = −0.459, *p* < 0.001). Significant negative correlations were also observed for the 2–5 mm shell (shell-2) (ρ = −0.318, *p* = 0.009) and the 5–10 mm shell (shell-3) (ρ = −0.301, *p* = 0.013). Conversely, no significant associations were found between peritumoral ADC values and the Ki-67 proliferation index across any peritumoral layer (all *p* > 0.05).

### 3.9. Interobserver Reproducibility of ADC-Based Measurements

High interobserver agreement was observed for intratumoral ADC measurements (ICC = 0.79). For peritumoral ADC measurements, the ICC values were 0.76 for the 0–2 mm shell (shell-1), 0.74 for the 2–5 mm shell (shell-2), and 0.77 for the 5–10 mm shell (shell-3).

With respect to normalized ADC parameters, the ICC was 0.78 for normalized intratumoral ADC (intratumoral rADC), 0.75 for normalized shell-1 rADC (0–2 mm), 0.73 for normalized shell-2 rADC (2–5 mm), and 0.76 for normalized shell-3 rADC (5–10 mm). Similarly, interobserver agreement for ratio-based parameters was acceptable, with ICC values of 0.80 for the intratumoral-to-peritumoral ADC ratio (0–2 mm shell), 0.75 for the 2–5 mm shell, and 0.78 for the 5–10 mm shell.

These findings indicate that the semi-automated VOI-based measurement approach used in this study demonstrates good interobserver reliability and satisfactory measurement reproducibility.

## 4. Discussion

In this study, we present a comprehensive evaluation of the relationships between diffusion-based quantitative parameters and tumor biology, as well as regional dissemination potential in invasive breast cancer. Our findings indicate that tumor biology is not solely determined by intratumoral cellular characteristics; rather, microenvironmental processes within the immediately adjacent peritumoral zone (0–2 mm) may play a role in biological aggressiveness and early regional spread [4]. In this context, assessing intratumoral diffusion properties relative to the peritumoral microenvironment may provide a conceptual framework for the evaluation of associations with clinically relevant outcomes, such as axillary lymph node metastasis. This ratio-based approach integrates cell-dominant intratumoral biology with the adjacent peritumoral microenvironment—encompassing stromal, lymphatic, and microvascular remodeling—within a unified quantitative framework, thereby offering a potentially more sensitive reflection of biological aggressiveness and early regional dissemination in breast cancer. Moreover, the consistent inverse associations observed between intratumoral ADC values and axillary lymph node metastasis, lymphovascular invasion, histologic grade, and the Ki-67 proliferation index further support diffusion restriction as a fundamental imaging surrogate of biological aggressiveness in breast cancer [19]. Collectively, these findings suggest that evaluating peritumoral diffusion characteristics in contextual relation to intratumoral tissue, rather than relying solely on absolute ADC measurements, may provide additional insights that could potentially enhance the clinical interpretability of noninvasive imaging biomarkers in breast cancer.

Evaluating model performance at clinically meaningful operating points provides a more applicable framework for preoperative risk stratification. In this context, the intratumoral/peritumoral ADC ratio (0–2 mm shell-1) appears to offer potential value in identifying patients with a higher likelihood of axillary lymph node metastasis under rule-in conditions, where higher specificity is prioritized. Conversely, its relatively limited specificity at higher sensitivity levels suggests that it may be less suitable for rule-out purposes. This distinction highlights the importance of aligning imaging biomarkers with specific clinical use scenarios rather than relying solely on global performance metrics. Furthermore, the observed increase in model performance when the ADC ratio is considered alongside tumor size supports its role as a complementary parameter within a multivariable framework. Rather than replacing conventional predictors, this parameter may provide additional, microenvironment-informed information that refines risk estimation in the preoperative setting. Taken together, these findings support a conceptual shift from viewing ADC-based metrics as standalone predictors toward their integration into multiparametric, clinically oriented decision models. Such an approach may better reflect the complex biological and microenvironmental processes underlying tumor dissemination and may contribute to the translational interpretation of diffusion-based imaging biomarkers.

From a clinical decision-making perspective, the present findings highlight the importance of aligning threshold selection with the intended clinical application. While the Youden index provides a statistically optimal cut-off, it does not necessarily reflect clinically meaningful decision thresholds. In the context of preoperative risk stratification, where false positive findings may lead to unnecessary invasive procedures or overtreatment, a rule-in strategy prioritizing specificity is more appropriate. In this regard, the use of predefined clinical operating points (Table 4) allows for a simplified representation of potential decision scenarios, whereas the threshold-dependent analysis based on ROC-derived cut-off values (Table 6) provides a more detailed characterization of the trade-off between sensitivity and specificity across clinically relevant thresholds. Together, these complementary approaches offer a more comprehensive framework for interpreting diagnostic performance. Our results demonstrate that the intratumoral/peritumoral ADC ratio shows moderate performance at specificity-oriented thresholds, supporting its potential role in identifying patients with a higher likelihood of axillary lymph node metastasis. However, its limited specificity at high sensitivity levels suggests that it is not suitable as a rule-out biomarker. Accordingly, the proposed parameter is better positioned as a complementary rule-in imaging marker rather than a standalone screening tool. Importantly, although bootstrap analysis demonstrated acceptable internal stability of cut-off values, these thresholds remain data-dependent and may be subject to optimism bias, particularly when derived and evaluated within the same dataset. This limitation is consistent with the modest difference observed between apparent and bootstrap-corrected model performance. Therefore, the proposed thresholds should be interpreted with caution, and external validation in independent cohorts is necessary before clinical implementation. Overall, these findings support a shift from reliance on single, statistically derived cut-offs toward a more flexible, clinically oriented threshold framework that reflects real-world decision-making and facilitates the integration of imaging biomarkers into multiparametric risk assessment models.

Regarding the intended clinical scenario, the proposed peritumoral multilayered shell model may serve as a complementary rule-in imaging parameter, particularly in equivocal clinical situations. For example, in cases where routine MRI features (such as tumor size or morphology) do not clearly suggest a higher-risk profile, a relatively low intratumoral-to-peritumoral ADC ratio or diffusion characteristics within the 0–2 mm ‘invasion front’ may provide additional supportive information regarding the likelihood of nodal involvement. Such information may contribute to a more refined preoperative risk assessment when interpreted alongside established clinical and imaging findings. In this context, the parameter may be considered an adjunct to existing evaluation strategies rather than a determinant of clinical decision-making. By acting as a complementary imaging marker, this quantitative approach may help reduce diagnostic uncertainty in cases where standard qualitative assessments remain inconclusive.

Our findings suggest that the peritumoral region represents a dynamic microenvironment rather than a biologically uniform compartment, with properties that vary as a function of distance from the tumor margin [13,18]. In the present study, absolute peritumoral ADC values alone showed limited discriminative ability, whereas ratio-based metrics demonstrated more consistent associations with biological and dissemination-related parameters. In particular, the immediately adjacent 0–2 mm peritumoral zone corresponds to the invasion front, where interactions between tumor cells and stromal cells, lymphatic channels, and microvascular structures are most intense; conversely, the influence of the tumor is known to progressively attenuate with increasing distance from the tumor boundary [13,18,20,21]. This spatial biological gradient is also reflected in diffusion characteristics, as ADC values measured within narrow peritumoral zones adjacent to the invasion front appear more sensitive to stromal remodeling, increased vascular permeability, and alterations in interstitial fluid dynamics [15]. Accordingly, conventional approaches that define the peritumoral region as a single homogeneous ROI may dilute biologically relevant signals by sampling more distant parenchyma where tumor influence is diminished [15]. In contrast, the distance-dependent concentric shell approach accounts for this gradual biological transition within the peritumoral microenvironment, enabling more selective evaluation of regions where tumor–stroma interactions are most pronounced. Through intratumoral-to-peritumoral ADC ratios in particular, this method integrates cellularity-dominant intratumoral tissue with microenvironment-specific peritumoral alterations along a unified quantitative axis [22,23]. Unlike many previous peritumoral ADC studies that rely on a single ROI surrounding the tumor margin, the present study employs a distance-dependent multilayered shell approach to characterize diffusion changes across distinct spatial zones surrounding the tumor. This design enables more detailed evaluation of the tumor–microenvironment interface and captures the transition from the immediate tumor–stroma interface (0–2 mm) to a more distal stromal remodeling zone (2–5 mm), which may involve varying degrees of desmoplasia, immune cell infiltration, and microvascular alterations. Interestingly, the lowest median peritumoral ADC values were observed in the 2–5 mm shell rather than in the immediately adjacent 0–2 mm invasion front. This finding suggests that the biologically most diffusion-restricted peritumoral zone may not fully overlap with the region showing the strongest imaging associations. While the 2–5 mm shell may correspond to a desmoplastic stromal remodeling zone characterized by increased cellularity and fibrosis, the immediately adjacent 0–2 mm shell likely reflects the invasion front, where tumor–stroma, lymphatic, and microvascular interactions are most intense. Accordingly, the strongest associations with lymphovascular invasion and axillary nodal metastasis observed in the 0–2 mm shell suggest that imaging-based assessment of dissemination risk may be more closely related to tumor–microenvironment interaction at the invasion front than by the degree of diffusion restriction alone. Interestingly, our results suggest that spatial differentiation of the peritumoral region may help to better delineate biologically relevant stromal signals from potential confounding effects such as peritumoral edema and partial-volume uncertainties that may influence measurements within the immediately adjacent 0–2 mm shell. This finding supports the value of distance-sensitive diffusion analysis for improving the biological interpretability of peritumoral imaging biomarkers.

The use of a normalized rADC approach in our study reflects a deliberate methodological choice aimed at strengthening the biological interpretability of diffusion measurements. Absolute ADC values are influenced by multiple technical and patient-related factors, including magnetic field strength, b-value selection, sequence parameters, and interindividual variations in breast parenchymal characteristics [9,11]. Such variability may obscure biologically relevant signals, particularly in retrospective or multicenter studies [19]. In contrast, rADC values calculated using contralateral normal fibroglandular tissue as a reference partially compensate for systemic and parenchymal diffusion characteristics, thereby enhancing the relative and within-subject comparability of measurements [20]. In our study, the observation that rADC-based intratumoral and ratio-based parameters exhibited more consistent discriminative patterns with respect to axillary lymph node metastasis and lymphovascular invasion than absolute ADC measurements suggests that this normalization strategy amplifies biologically meaningful signals [15]. These findings support the notion that rADC is not merely a technical correction to reduce measurement variability, but rather a functionally relevant imaging biomarker that reflects the relative diffusion behavior of tumor tissue in relation to its surrounding parenchyma, thereby reinforcing its potential clinical applicability [9,11].

Previous research has suggested that peritumoral diffusion measurements in breast cancer (peritumoral ADC) may reflect not only the surrounding tissue but also microenvironmental processes adjacent to the invasion front and may therefore be associated with lymphovascular invasion (LVI) and aggressive biological tumor features [13,18]. In particular, Okuma et al. demonstrated that the peritumoral-to-tumor ADC ratio may be more informative than intratumoral ADC alone for predicting LVI and other aggressive biological characteristics, highlighting the conceptual advantage of a ratio-based approach [15]. Similarly, Igarashi et al. reported associations between tumor ADC, peritumoral ADC, and the peritumoral-to-tumor ADC ratio and LVI, further supporting the clinical relevance of the peritumoral component [21]. However, a major methodological challenge in this field remains the lack of standardization regarding the location and width of the peritumoral region used for ADC measurement. Kettunen et al. demonstrated that different ROI selection strategies can substantially alter peritumoral ADC results, thereby limiting comparability across studies [15]. To partially address this limitation, our study sampled the peritumoral region using distance-sensitive multilayered shells (0–2 mm, 2–5 mm, and 5–10 mm) rather than a single homogeneous band and further applied normalization to contralateral fibroglandular tissue (rADC) to reduce technical and parenchymal variability. Within this framework, the observation that ratio-based metrics—particularly within shells closest to the tumor—exhibited more consistent associations with regional dissemination phenotypes and provided the most pronounced discrimination for axillary lymph node status suggests that the combined shell-based and normalization approach may more effectively capture biologically relevant signals. Moreover, the findings of lower intratumoral ADC and reduced tumor-to-0–2 mm peritumoral ADC ratios in LVI-positive cases mirror the biological pattern reported by Okuma et al., reinforcing the validity of a ratio-based strategy [15]. In addition, the LVI analysis in the present study further strengthened the biological interpretation of diffusion-based tumor characterization. Tumors with lymphovascular invasion demonstrated significantly lower intratumoral ADC and rADC values, indicating more pronounced diffusion restriction consistent with higher cellular density and aggressive tumor behavior. Importantly, the intratumoral-to-peritumoral ADC ratio in the immediate 0–2 mm shell was also significantly reduced in LVI-positive tumors. This finding may suggest that the diffusion contrast between the tumor and its immediate microenvironment becomes attenuated in the presence of lymphovascular invasion, potentially reflecting early tumor–stroma interaction and microvascular infiltration at the invasion front. In contrast, diffusion metrics derived from more distant peritumoral shells did not show significant differences, highlighting the biological importance of the immediate tumor boundary. Taken together, our study extends prior work by incorporating peritumoral diffusion measurements within a distance-sensitive and normalized framework, thereby situating previously described biological associations within a more systematic and clinically relevant context.

The predictive analyses further supported the clinical relevance of this approach. Among all evaluated diffusion parameters, the intratumoral-to-peritumoral ADC ratio in the 0–2 mm shell demonstrated the numerically highest AUC among the evaluated diffusion parameters for axillary lymph node metastasis in both ROC and logistic regression analyses. This finding further supports the potential importance of the immediate peritumoral zone for imaging-based assessment of early regional dissemination risk. These predictive findings should be interpreted as exploratory and hypothesis-generating rather than as evidence of immediate clinical applicability. The moderate AUC values and the absence of external validation limit the use of these metrics as standalone predictive tools. Future studies incorporating larger cohorts and external validation are required before clinical implementation can be considered. Given the exploratory design of the present study and the moderate level of discriminative performance, these findings should be interpreted with appropriate caution.

In interpreting these predictive findings, an important methodological consideration relates to the sensitivity of the proposed approach to segmentation variability and boundary definition. Although semi-automated volumetric segmentation with manual refinement was applied to standardize tumor delineation, the initial definition of tumor boundaries remains partially operator-dependent. This dependency becomes particularly relevant in the context of narrow peritumoral shells, especially in the 0–2 mm and 2–5 mm layers, where even minor variations in boundary positioning may lead to measurable differences in voxel inclusion and derived diffusion metrics. In addition, partial volume effects represent a non-negligible source of measurement uncertainty. Given that the thickness of the innermost peritumoral shells may approach or fall below the intrinsic spatial resolution of diffusion-weighted imaging, ADC values in these regions may reflect mixed tissue signals at the tumor–parenchyma interface. To mitigate these effects, several methodological strategies were systematically implemented, including isotropic resampling, morphological boundary erosion, automated shell generation, and voxel-wise volumetric averaging. Nevertheless, despite these precautions, residual bias related to segmentation uncertainty and boundary definition cannot be entirely eliminated. The interobserver agreement analysis demonstrated good reproducibility of both intratumoral and peritumoral measurements; however, it is important to emphasize that ICC primarily reflects agreement between observers and does not fully capture robustness against segmentation perturbations or test–retest variability. The absence of dedicated robustness analyses—such as segmentation perturbation experiments or repeatability assessments—therefore represents an important methodological limitation that may influence the stability of ratio-based parameters, particularly in thin peritumoral regions. These considerations suggest that the reported diffusion metrics, especially the intratumoral/peritumoral ADC ratio (0–2 mm shell-1), should not be interpreted as fixed or absolute quantitative thresholds, but rather as biologically informed, context-dependent indicators. Accordingly, the observed associations should be interpreted with appropriate caution, acknowledging the potential influence of segmentation-related variability on measurement precision. At the same time, the consistent direction and pattern of associations across multiple biological endpoints, together with the preservation of discriminative performance in the temporally independent validation cohort, support the biological and methodological coherence of the findings, suggesting that they are not solely driven by measurement noise. Within this framework, the present study should be considered robust in its analytical design yet limited by inherent methodological constraints related to segmentation sensitivity. The findings are therefore best positioned within an exploratory and hypothesis-generating context, providing preliminary but meaningful insights into tumor–microenvironment interactions and their potential imaging correlates. Future studies incorporating systematic robustness analyses, including segmentation perturbation frameworks, test–retest datasets, and fully automated or artificial intelligence-based segmentation approaches, will be essential to reduce operator dependency and to establish the reproducibility, stability, and clinical translatability of these imaging-derived biomarkers.

In addition, the present study involves multiple statistical comparisons across a broad range of diffusion-derived parameters and biological endpoints, which inherently increases the risk of type I error. Although the analyses were conducted within a hypothesis-driven framework, no formal correction for multiple comparisons was applied. This methodological choice reflects the exploratory nature of the study, aiming to identify potentially relevant imaging–biological associations rather than to establish definitive inferential conclusions. Accordingly, the reported findings—particularly those derived from subgroup analyses or parameters with borderline statistical significance—should be interpreted with caution. While the consistency of observed associations across multiple endpoints and the supportive results from internal validation analyses partially mitigate concerns regarding false positive findings, these approaches do not fully eliminate this risk. Therefore, the present results should be considered hypothesis-generating, and future studies incorporating pre-specified statistical correction strategies and external validation are warranted to confirm the robustness and reproducibility of these findings.

To further address generalizability, a temporally independent validation cohort was analyzed. The preservation of AUC values and the maintenance of specificity at clinically relevant operating points support the robustness of the intratumoral/peritumoral ADC ratio (0–2 mm shell-1). Importantly, the direction of association between the intratumoral/peritumoral ADC ratio (0–2 mm shell-1) and axillary lymph node status remained consistent across cohorts, suggesting reproducibility of the underlying biological signal. However, this validation was performed within a single institution and involved a relatively limited sample size, which may constrain the full assessment of generalizability across different populations, imaging protocols, and clinical settings. Therefore, these findings should be interpreted as preliminary yet methodologically supported evidence of generalizability rather than definitive proof. In line with this, the present results should be considered exploratory and hypothesis-generating, providing an initial indication of the potential clinical relevance of the intratumoral/peritumoral ADC ratio (0–2 mm shell-1). The use of a temporally independent cohort, combined with the application of an unchanged analysis pipeline and predefined thresholds without re-optimization, reduces the risk of data leakage and strengthens the internal–external validity continuum of the present findings. Nevertheless, broader validation across independent multicenter datasets with heterogeneous imaging conditions and patient populations is required to confirm the stability and transportability of the intratumoral/peritumoral ADC ratio (0–2 mm shell-1). Accordingly, validation across independent multicenter cohorts with diverse imaging protocols and patient populations remains essential to establish the generalizability and robustness of these findings before clinical translation or routine clinical implementation can be considered. In this context, the intratumoral/peritumoral ADC ratio (0–2 mm shell-1) should be interpreted as having potential as an imaging biomarker, rather than a standalone diagnostic tool, and may provide complementary, microenvironment-informed information when integrated into multiparametric clinical decision-making frameworks.

Our findings regarding molecular subtypes and immunohistochemical markers suggest that peritumoral diffusion characteristics may provide complementary and context-dependent information reflecting tumor biology [4,7,12,24,25]. However, these results should be interpreted with caution, particularly in subgroup analyses involving relatively small sample sizes. Although peritumoral rADC values did not demonstrate statistically significant differences across molecular subtypes, a trend toward higher values in biologically aggressive subtypes, such as HER2-enriched and triple-negative breast cancer, was observed. Given the limited number of cases in these subgroups and the multiple comparisons performed, such trend-level findings may be susceptible to statistical instability and false positive results, and therefore should not be overinterpreted. The observed patterns may tentatively reflect microenvironmental processes, including stromal remodeling, increased microvascular permeability, and interstitial edema; however, these interpretations remain speculative and require further validation. Similarly, the higher intratumoral ADC and ratio-based parameters observed in the luminal A subtype may suggest differences in tumor cellularity and microenvironmental interaction, but these findings should be considered hypothesis-generating rather than definitive. Furthermore, the associations identified between the Ki-67 proliferation index and diffusion-based parameters should be interpreted within the same exploratory framework. While these findings are biologically plausible, the absence of formal multiple comparison correction and the potential for type I error necessitate cautious interpretation. Taken together, these observations suggest that peritumoral ADC measurements may have potential as functional imaging indicators related to tumor biology; however, they should not be considered conclusive. Future studies with larger and more balanced subtype distributions, predefined statistical correction strategies, and independent validation cohorts are required to confirm the robustness and reproducibility of these associations [26].

The findings of this study suggest that peritumoral rADC measurements may provide potentially clinically relevant meaningful incremental information in the preoperative breast MRI assessment [5,11]. These noninvasively derived quantitative parameters, obtainable in the preoperative setting, may provide preliminary insight into the risk of axillary lymph node metastasis, thereby contributing to a more refined evaluation of axillary risk, particularly in cases with equivocal clinical and imaging findings [14,23]. In this context, rADC-based measurements reflecting the peritumoral microenvironment may have potential exploratory utility as a supportive tool for the risk stratification of axillary involvement prior to sentinel lymph node biopsy in selected patients [6,8]. Moreover, the early identification of diffusion patterns associated with more aggressive biological behavior may help guide closer surveillance strategies or prompt earlier intensification of systemic treatment approaches [7,20]. Accordingly, when integrated with existing clinical, histopathological, and imaging data, peritumoral rADC may be considered a potential complementary, noninvasive imaging biomarker that may contribute personalized treatment planning in breast cancer [5,11].

The principal strengths of this study include the evaluation of the peritumoral region using a novel multilayered shell approach that accounts for biological heterogeneity, the incorporation of normalized rADC measurements referenced to contralateral normal fibroglandular tissue, and the integrated assessment of intratumoral diffusion characteristics together with the peritumoral microenvironment [9,15]. Correlating imaging-derived parameters with pathological gold standards—such as immunohistochemical markers, histologic grade, lymphovascular invasion, and axillary lymph node status—further enhances the biological and clinical validity of the findings. In addition, the good intraobserver agreement observed for both intratumoral and peritumoral ADC measurements, as demonstrated by ICC analysis, supports the reliability and reproducibility of the proposed shell-based approach despite the use of semi-automated segmentation [11].

Several limitations of this study should be acknowledged. First, its single-center, retrospective design may have introduced selection bias and limited the generalizability of the findings. In addition, the relatively modest sample size and the small number of cases in certain molecular subtypes (particularly HER2-enriched and triple-negative breast cancers) may have reduced statistical power, particularly in subgroup analyses, and may increase the risk of unstable estimates. Owing to the inherent characteristics of DWI, the limited spatial resolution and susceptibility-related distortions may have affected measurement accuracy, especially in small lesions and in the precise evaluation of peritumoral regions [9,11]. Because the thickness of the innermost peritumoral shell (0–2 mm) is smaller than the original slice thickness of the diffusion-weighted imaging sequence, partial-volume effects cannot be entirely excluded. Although isotropic resampling and morphological erosion were implemented to minimize boundary-related signal contamination, the biological interpretation of diffusion characteristics within the immediate peritumoral “invasion front” should be considered with appropriate caution. In addition, because multilayer peritumoral shells may partially overlap with surrounding adipose tissue in the breast, complete exclusion of fat-containing voxels cannot be guaranteed in semi-automated segmentation workflows. Although normalization to contralateral fibroglandular tissue and voxel-wise volumetric averaging were used to minimize this effect, the potential influence of adipose tissue on peritumoral ADC measurements should be considered when interpreting these results. Furthermore, the spatial distribution of peritumoral ADC values should be interpreted with caution. The lowest median ADC was observed in the 2–5 mm shell rather than in the immediately adjacent 0–2 mm invasion front, which may appear to diverge from the expected distance-dependent gradient of tumor influence. This pattern likely reflects the complex biological heterogeneity of the peritumoral microenvironment. The immediately adjacent 0–2 mm zone may be influenced by increased vascular permeability, lymphatic obstruction, and interstitial edema, which can increase extracellular water content and elevate ADC values, whereas the 2–5 mm region may correspond to a desmoplastic stromal remodeling zone characterized by increased cellularity and fibrosis, resulting in more restricted diffusion. In addition, methodological factors may have contributed to this observation. The narrow thickness of the 0–2 mm shell makes it particularly susceptible to partial-volume effects and segmentation boundary uncertainties, and smoothing of tumor borders during VOI generation may have resulted in the most diffusion-restricted tissue being partially included within the intratumoral volume. Because the proposed analysis relied on narrow peritumoral shells, particularly in the 0–2 mm and 2–5 mm regions, small variations in segmentation boundaries may have influenced the measured ADC values and derived ratio-based parameters. Although automated shell generation and interobserver reproducibility analysis were used to improve measurement reliability, more extensive reader-variability analyses and fully automated segmentation approaches may further strengthen methodological robustness in future studies. Finally, the intratumoral and peritumoral ROI/VOI segmentation was primarily performed by a single radiologist and relied in part on manual procedures, which introduces the potential for reader-dependent bias. Although interobserver agreement analysis demonstrated good reproducibility, this was performed in a subset of cases, and the results should therefore be interpreted with appropriate caution. Despite the good interobserver reproducibility observed for the ADC measurements, additional robustness assessments—such as test–retest reproducibility or segmentation perturbation analysis—were not performed. Future studies incorporating dedicated test–retest imaging datasets and automated segmentation approaches may provide further insight into the robustness and stability of these imaging-derived parameters. In addition, the present study involved multiple statistical comparisons across a broad set of ADC-derived metrics and biological variables. Although the analyses were performed in a hypothesis-driven framework, the number of comparisons increases the potential risk of type I error. No formal correction for multiple comparisons was applied, which may further increase the likelihood of false positive findings. Although internal validation using repeated cross-validation and bootstrap resampling demonstrated stable performance estimates, no external validation cohort was available; therefore, the generalizability of these findings should be confirmed in larger independent multicenter datasets. Therefore, the reported findings—particularly those derived from subgroup and exploratory analyses—should be interpreted with appropriate caution. These results should be considered hypothesis-generating and warrant confirmation in larger, prospective, and preferably multicenter cohorts with pre-specified statistical correction strategies.

Future studies should include larger, multicenter patient cohorts to validate the present findings and facilitate their translation into clinical practice. The adoption of automated or artificial intelligence-based peritumoral segmentation approaches may reduce observer-dependent variability and further strengthen methodological standardization [11]. Moreover, future segmentation frameworks incorporating automated tissue-classification algorithms capable of distinguishing fibroglandular tissue from adipose tissue at the voxel level may further improve the tissue specificity and biological interpretability of peritumoral diffusion measurements. In addition, the integration of advanced diffusion models—such as intravoxel incoherent motion (IVIM) and diffusion kurtosis imaging (DKI)—may enable more detailed characterization of microvascular and microstructural features within the peritumoral microenvironment [10,19]. Finally, investigating the associations between peritumoral rADC-based parameters and prognostic outcomes, including long-term survival, recurrence, and treatment response, will be critical to establishing the true clinical value of these imaging biomarkers [13].

## 5. Conclusions

This study demonstrates that a multilayered peritumoral shell approach combined with rADC measurements normalized to normal fibroglandular tissue captures imaging patterns associated with biological aggressiveness and axillary lymph node status in breast cancer. Evaluating intratumoral diffusion characteristics in conjunction with the peritumoral microenvironment highlights that tumor biology is shaped not only by cellular components but also by surrounding stromal and vascular conditions. The proposed approach may be considered for incorporation into breast MRI analysis without additional imaging sequences and may provide complementary information for risk assessment when interpreted within a multiparametric framework, pending validation in larger prospective studies.

## Figures and Tables

**Table 1:** Baseline demographic and pathological characteristics of study population.

Variable	Value
Age, years	
Mean ± SD	48.5 ± 13.6
Median (range)	45.5 (24–81)
Tumor volume, mm^3^	
Median (IQR)	3999 (1123–9618)
Histological subtype, *n* (%)	
Invasive ductal carcinoma	47 (69.1)
Invasive lobular carcinoma	8 (11.8)
Apocrine invasive carcinoma	3 (4.4)
Tubular carcinoma	3 (4.4)
Mucinous carcinoma	2 (2.9)
Mixed mucinous + invasive lobular carcinoma	1 (1.5)
Mixed invasive ductal + invasive lobular carcinoma	1 (1.5)
Papillary carcinoma	1 (1.5)
Micropapillary carcinoma	1 (1.5)
Malignant mesenchymal tumor	1 (1.5)
Histological grade (Nottingham), *n* (%)	
Grade 1	22 (32.4)
Grade 2	35 (51.5)
Grade 3	11 (16.2)
Associated in situ component, *n* (%)	
DCIS present	24 (35.3)
LCIS present	4 (5.9)
Immunohistochemical markers, *n* (%)	
Estrogen receptor (ER)-positive	57 (83.8)
Progesterone receptor (PR)-positive	54 (79.4)
HER2-positive	36 (52.9)
Ki-67 proliferation index	
Median (IQR)	0.25 (0.15–0.43)
Molecular subtype, *n* (%)	
Luminal A	13 (19.1)
Luminal B (HER2-negative)	28 (41.2)
Luminal B (HER2-positive)	16 (23.5)
HER2-enriched (non-luminal)	6 (8.8)
Triple-negative breast cancer	5 (7.4)
Invasion characteristics, *n* (%)	
Lymphovascular invasion (LVI)-positive	17 (25.0)
Perineural invasion-positive	14 (20.6)
Axillary lymph node status, *n* (%)	
Positive	27 (39.7)
Negative	41 (60.3)

Data are presented as mean ± standard deviation, median (interquartile range), or number of patients (percentage), as appropriate. Abbreviations: DCIS, ductal carcinoma in situ; LCIS, lobular carcinoma in situ; ER, estrogen receptor; PR, progesterone receptor; HER2, human epidermal growth factor receptor 2; IQR, interquartile range; LVI, lymphovascular invasion.

**Table 2:** Summary of intratumoral, peritumoral, reference, normalized, and ratio-based ADC metrics.

ADC Parameter	Median (IQR)
Intratumoral ADC	0.88 (0.75–1.12)
Peritumoral ADC (0–2 mm shell-1)	1.52 (1.43–1.67)
Peritumoral ADC (2–5 mm shell-2)	1.42 (1.27–1.56)
Peritumoral ADC (5–10 mm shell-3)	1.57 (1.47–1.77)
Reference ADC (contralateral fibroglandular tissue)	1.55 (1.50–1.69)
Normalized ADC (Intratumoral_rADC)	0.57 (0.49–0.71)
Normalized ADC (Shell-1_rADC, 0–2 mm)	0.97 (0.90–1.05)
Normalized ADC (Shell-2_rADC, 2–5 mm)	0.91 (0.78–0.97)
Normalized ADC (Shell-3_rADC, 5–10 mm)	0.99 (0.89–1.11)
Intratumoral/peritumoral ADC ratio (0–2 mm shell-1)	0.59 (0.49–0.74)
Intratumoral/peritumoral ADC ratio (2–5 mm shell-2)	0.66 (0.55–0.83)
Intratumoral/peritumoral ADC ratio (5–10 mm shell-3)	0.55 (0.48–0.73)

ADC values are expressed as ×10^−3^ mm^2^/s. Data are presented as the median (interquartile range). Normalized ADC values represent ratios relative to the reference ADC obtained from contralateral fibroglandular tissue. Tumor/peritumoral ADC ratios were calculated by dividing intratumoral ADC values by the corresponding peritumoral shell ADC values on a per-patient basis.

**Table 3:** Comparison of ADC, rADC, and intratumoral/peritumoral ADC ratios between pN0 and pN+.

ADC Parameter	pN− (*n* = 41) Median (IQR)	pN+ (*n* = 27) Median (IQR)	*p* Value
Intratumoral ADC (×10^−3^ mm^2^/s)	0.94 (0.81–1.12)	0.75 (0.715–0.93)	0.0037
Peritumoral ADC (0–2 mm shell-1)	1.49 (1.43–1.62)	1.62 (1.44–1.78)	0.0966
Peritumoral ADC (2–5 mm shell-2)	1.39 (1.31–1.54)	1.48 (1.23–1.67)	0.4295
Peritumoral ADC (5–10 mm shell-3)	1.56 (1.48–1.66)	1.72 (1.42–1.83)	0.1277
Normalized ADC (Intratumoral_rADC)	0.61 (0.52–0.74)	0.51 (0.45–0.62)	0.0145
Normalized ADC (Shell-1_rADC, 0–2 mm)	0.94 (0.89–1.03)	1.03 (0.90–1.09)	0.0772
Normalized ADC (Shell-2_rADC, 2–5 mm)	0.90 (0.79–0.96)	0.91 (0.78–1.12)	0.5986
Normalized ADC (Shell-3_rADC, 5–10 mm)	0.97 (0.89–1.06)	1.09 (0.88–1.17)	0.0930
Intratumoral/peritumoral ADC ratio (0–2 mm shell-1)	0.63 (0.52–0.76)	0.50 (0.45–0.62)	0.0066
Intratumoral/peritumoral ADC ratio (2–5 mm shell-2)	0.70 (0.59–0.84)	0.61 (0.52–0.72)	0.0802
Intratumoral/peritumoral ADC ratio (5–10 mm shell-3)	0.60 (0.50–0.75)	0.48 (0.41–0.61)	0.0089

ADC values are expressed as ×10^−3^ mm^2^/s. Continuous variables are presented as the median (interquartile range, IQR). rADC values represent normalized ADC parameters calculated as the ratio of each ADC measurement to the reference ADC obtained from contralateral fibroglandular breast tissue on a per-patient basis. Intratumoral/peritumoral ADC ratios were calculated by dividing intratumoral ADC values by the corresponding peritumoral shell ADC values (0–2 mm, 2–5 mm, and 5–10 mm) for each patient. Group comparisons between pN0 and pN+ were performed using the Mann–Whitney U test. *p*-values < 0.05 were considered statistically significant.

**Table 4:** Decision-oriented performance metrics of the intratumoral/peritumoral ADC ratio (0–2 mm shell-1) for predicting axillary lymph node metastasis.

Metric	Operating Point	Sensitivity	Specificity	Clinical Interpretation
Intratumoral/peritumoral ADC ratio (0–2 mm shell-1)	Specificity fixed at 80%	55.6**%**	80**%**	Rule-in utility (moderate)
Intratumoral/peritumoral ADC ratio (0–2 mm shell-1)	Sensitivity fixed at 80%	80**%**	36.6**%**	Limited rule-out performance

Decision-oriented performance metrics were calculated at predefined operating points. Sensitivity and specificity are presented as percentages. The intratumoral/peritumoral ADC ratio (shell-1, 0–2 mm) represents the ratio of intratumoral ADC to the corresponding peritumoral shell ADC, calculated on a per-patient basis. These operating points illustrate clinically relevant decision thresholds and do not necessarily correspond to ROC-derived cut-off values (see Table 6).

**Table 5:** ROC summary of best ADC-based metrics for predicting axillary lymph node positivity (pN+).

Metric	AUC (95% CI)	*p*-Value (vs. 0.5)	Rule for Predicting pN+	Youden Index Cut-Off	Sensitivity	Specificity
Intratumoral/peritumoral ADC ratio (0–2 mm shell-1)	0.725 (0.590–0.841)	<0.001	≤Cut-off	0.552	66.7%	78.0%
Intratumoral ADC (×10^−3^ mm^2^/s)	0.710 (0.572–0.833)	0.002	≤Cut-off	0.760	59.3%	87.8%
Normalized ADC (Intratumoral_rADC)	0.677 (0.537–0.801)	0.011	≤Cut-off	0.558	70.4%	65.9%
Peritumoral ADC (0–2 mm shell-1)	0.634 (0.492–0.760)	0.068	≥Cut-off	0.963	70.4%	58.5%

The area under the ROC curve (AUC) is reported with 95% confidence intervals (CIs). *p*-values indicate whether the AUC differs significantly from chance level (AUC = 0.5). The cut-off values were determined using the Youden index. The sensitivity and specificity are presented as percentages. For metrics with a “≤cut-off” rule, values equal to or below the specified cut-off indicate a higher probability of axillary lymph node positivity (pN+), whereas for metrics with a “≥cut-off” rule, values equal to or above the cut-off indicate a higher probability of pN+. Tumor/Shell-1 ratio represents the ratio of intratumoral ADC to the ADC of the 0–2 mm peritumoral shell, calculated on a per-patient basis. Tumor_rADC and Shell-1_rADC represent normalized ADC values relative to the reference ADC obtained from contralateral fibroglandular breast tissue.

**Table 6:** Threshold-dependent diagnostic performance and bootstrap stability of the intratumor-al/peritumoral ADC ratio (0–2 mm shell-1) for predicting axillary lymph node metastasis.

Threshold Strategy	Cut-Off	Sensitivity	Specificity	PPV	NPV	Accuracy	Bootstrap Median Cut-Off	IQR of Cut-Off	Clinical İnterpretation
Youden index	0.552	66.7**%**	78.0**%**	66.7**%**	78.0**%**	73.5**%**	0.552	0.515–0.552	Balanced but not clinically optimized
Specificity ≈ 80%	0.515	59.3**%**	80.5**%**	66.7**%**	75.0**%**	70.6**%**	0.515	0.506–0.552	Suitable for rule-in (moderate enrichment)
Specificity ≈ 85%	0.506	51.9**%**	85.4**%**	70.0**%**	72.9**%**	67.6**%**	0.493	0.448–0.511	Higher confidence rule-in
Specificity ≈ 90–95%	0.436	33.3**%**	95.1**%**	81.8**%**	68.4**%**	61.8**%**	0.448	0.436–0.463	High-confidence rule-in (low sensitivity)
Sensitivity ≈ 80%	0.709	81.5**%**	36.6**%**	45.8**%**	75.0**%**	58.8**%**	—	—	Not suitable for rule-out

Diagnostic performance metrics are presented across cut-off values derived from the receiver operating characteristic (ROC) curve. Each threshold represents a distinct operating point. Sensitivity, specificity, positive predictive value (PPV), negative predictive value (NPV), and accuracy are expressed as percentages. Bootstrap resampling was used to assess the stability of selected cut-off values, with median cut-offs and interquartile ranges (IQRs) reported. These thresholds are data-driven and support clinically oriented interpretation, particularly for specificity-prioritized (rule-in) applications.

**Table 7:** Multivariable model performance and incremental predictive value for axillary lymph node metastasis (pN+).

Metric	Model Definition	AUC	Clinical Interpretation
Model 1	Tumor size	0.655	Baseline model
Model 2	Tumor size +intratumoral/peritumoral ADC ratio (0–2 mm shell-1)	0.712	Incremental value (modest improvement)

AUC values represent the area under the receiver operating characteristic curve. Model 1 includes tumor size as a conventional predictor. Model 2 incorporates both tumor size and the intratumoral/peritumoral ADC ratio (0–2 mm shell-1). The observed increase in AUC reflects the incremental predictive contribution of the ADC-derived parameter.

**Table 8:** Logistic regression for axillary lymph node positivity (pN+).

**Univariable Logistic Regression**			
**Predictor**	**OR**	**95% CI**	***p* ** **Value**
Intratumoral ADC (×10^−3^ mm^2^/s) (per +0.1)	0.731	0.572–0.935	0.0125
Normalized ADC (Intratumoral_rADC) (per +0.1)	0.644	0.444–0.933	0.0199
Normalized ADC (Shell-1_rADC, 0–2 mm) (per +0.1)	1.459	0.969–2.197	0.0705
Normalized ADC (Shell-2_rADC, 2–5 mm) (per +0.1)	1.103	0.851–1.429	0.4590
Normalized ADC (Shell-3_rADC, 5–10 mm) (per +0.1)	1.338	0.939–1.907	0.1050
Intratumoral/peritumoral ADC ratio (0–2 mm shell-1) (per +0.1)	0.583	0.413–0.822	0.0019
Intratumoral/peritumoral ADC ratio (2–5 mm shell-2) (per +0.1)	0.782	0.611–1.001	0.0510
Intratumoral/peritumoral ADC ratio (5–10 mm shell-3) (per +0.1)	0.701	0.523–0.939	0.0174
**Multivariable Logistic Regression (Adjusted for Tumor Volume)**			
**Predictor**	**OR**	**95% CI**	***p* Value**
Intratumoral/peritumoral ADC ratio (0–2 mm shell-1 (per +0.1)	0.624	0.437–0.891	0.0095
Tumor volume (per doubling)	1.178	0.908–1.528	0.2183

Odds ratios (ORs) are presented with 95% confidence intervals (CIs). For ADC parameters, ORs are reported per 0.1 × 10^−3^ mm^2^/s increase. For rADC parameters and intratumoral/peritumoral ADC ratios, ORs are reported per 0.1 unit increase. Tumor volume was included in multivariable analysis on a log2 scale, corresponding to the effect of tumor volume doubling. Multivariable models were adjusted for tumor volume. *p*-values < 0.05 were considered statistically significant.

**Table 9:** Comparison of baseline characteristics and diagnostic performance between temporally independent validation cohort and the primary cohort.

Parameter	Temporally Independent Validation Cohort (n = 20)	Primary Cohort (n = 68)
Age (mean ± SD)	42.3 ± 11.3	48.5 ± 13.6
Tumor size (mean ± SD, mm)	28.5 ± 14.1	24.1 ± 15.8
pN status, n (+/−)	10/10	27/41
AUC (Ratio, 0–2 mm shell-1)	0.75	0.725
Sensitivity (%)	50.0%	55.6%
Specificity (%)	80.0%	78.0%
Cut-off	0.552	0.552

Abbreviations: ADC, apparent diffusion coefficient; AUC, area under the receiver operating characteristic curve; SD, standard deviation; pN, pathological axillary lymph node status. Notes: Sensitivity and specificity are reported at the predefined cut-off (0.552) derived from the primary cohort and applied to the temporally independent validation cohort without re-optimization. Tumor size represents the largest tumor diameter measured on MRI.

**Table 10:** Comparison of ADC metrics according to lymphovascular invasion (LVI) status.

Parameters	LVI− (Median, IQR)	LVI+ (Median, IQR)	*p* Value
**Intratumoral ADC (×10^−3^** **mm^2^/s)**	**0.91 (0.78–1.14)**	**0.76 (0.66–0.92)**	**0.022**
Peritumoral ADC (0–2 mm shell-1)	1.52 (1.44–1.66)	1.58 (1.43–1.69)	0.676
Peritumoral ADC (2–5 mm shell-2)	1.41 (1.27–1.55)	1.42 (1.31–1.57)	0.854
Peritumoral ADC (5–10 mm shell-3)	1.56 (1.46–1.75)	1.72 (1.51–1.84)	0.528
**Normalized ADC (Intratumoral_rADC)**	**0.59 (0.50–0.73)**	**0.50 (0.40–0.63)**	**0.041**
Normalized ADC (Shell-1 rADC, 0–2 mm)	0.99 (0.92–1.07)	1.03 (0.94–1.12)	0.314
Normalized ADC (Shell-2 rADC, 2–5 mm)	0.91 (0.82–1.00)	0.93 (0.80–1.02)	0.712
Normalized ADC (Shell-3 rADC, 5–10 mm)	1.01 (0.92–1.11)	1.10 (0.88–1.17)	0.418
Intratumoral/peritumoral ADC ratio (0–2 mm shell-1)	**0.61 (0.52–0.75)**	**0.48 (0.41–0.59)**	**0.022**
Intratumoral/peritumoral ADC ratio (2–5 mm shell-2)	0.68 (0.56–0.84)	0.54 (0.45–0.67)	0.100
Intratumoral/peritumoral ADC ratio (5–10 mm shell-3)	0.57 (0.48–0.73)	0.48 (0.39–0.60)	0.060

Continuous variables are presented as the median (interquartile range, IQR). ADC values are expressed as ×10^−3^ mm^2^/s. rADC values represent normalized ADC parameters calculated as the ratio of each ADC measurement to the reference ADC obtained from contralateral fibroglandular breast tissue on a per-patient basis. Intratumoral-to-peritumoral ADC ratios (VRI) were calculated using raw ADC values by dividing intratumoral ADC by the corresponding peritumoral shell ADC values. Ratio-based metrics (rADC and VRI) are unitless. Group comparisons were performed using the Mann–Whitney U test. Statistically significant *p*-values (*p* < 0.05) are shown in bold.

**Table 11:** Comparison of ADC/rADC metrics across molecular subtypes.

Parameters	Luminal A	Luminal B (Combined)	HER2-Enriched	TNBC	Overall *p*
Intratumoral ADC (×10^−3^ mm^2^/s)	1.24 (1.12–1.31)	0.88 (0.75–1.04)	0.71 (0.69–0.75)	0.77 (0.75–0.81)	<0.001
Peritumoral ADC (0–2 mm shell-1)	1.52 (1.49–1.68)	1.52 (1.42–1.70)	1.54 (1.48–1.64)	1.45 (1.32–1.57)	0.623
Peritumoral ADC (2–5 mm shell-2)	1.50 (1.39–1.59)	1.37 (1.26–1.54)	1.36 (1.13–1.71)	1.55 (1.14–1.63)	0.431
Peritumoral ADC (5–10 mm shell-3)	1.55 (1.48–1.66)	1.58 (1.47–1.77)	1.52 (1.37–1.62)	1.69 (1.63–1.78)	0.848
Normalized ADC (Intratumoral_rADC)	0.76 (0.58–0.86)	0.57 (0.50–0.65)	0.46 (0.43–0.51)	0.49 (0.49–0.53)	0.001
Normalized ADC (Shell-1_rADC, 0–2 mm)	0.95 (0.90–1.01)	0.99 (0.90–1.06)	1.03 (0.90–1.13)	0.92 (0.78–1.00)	0.560
Normalized ADC (Shell-2_rADC, 2–5 mm)	0.95 (0.84–0.98)	0.90 (0.79–0.97)	0.81 (0.70–1.07)	0.91 (0.75–0.96)	0.553
Normalized ADC (Shell-3_rADC, 5–10 mm)	0.96 (0.91–1.03)	1.04 (0.90–1.14)	0.96 (0.83–1.09)	0.99 (0.96–1.10)	0.881
Intratumoral/peritumoral ADC ratio (0–2 mm shell-1)	0.84 (0.61–0.88)	0.59 (0.46–0.70)	0.49 (0.47–0.51)	0.58 (0.52–0.64)	0.005
Intratumoral/peritumoral ADC ratio (2–5 mm shell-2)	0.89 (0.61–0.94)	0.65 (0.53–0.80)	0.56 (0.43–0.63)	0.71 (0.50–0.76)	0.034
Intratumoral/peritumoral ADC ratio (5–10 mm shell-3)	0.81 (0.64–0.95)	0.55 (0.47–0.69)	0.51 (0.40–0.54)	0.48 (0.47–0.73)	0.008

Values are shown as median (IQR). Tumor_ADC and Shell ADC values are in ×10^−3^ mm^2^/s. rADC and Tumor/Shell ratios are unitless. Overall *p*-values are from Kruskal–Wallis tests.

**Table 12:** Correlations between ADC-based diffusion parameters, histological grade, and Ki-67 proliferation index.

Parameter	Histological Grade (Spearman ρ)	*p* Value	Ki-67 Index (Spearman ρ)	*p* Value
Intratumoral ADC (×10^−3^ mm^2^/s)	−0.456	<0.001	−0.463	<0.001
Peritumoral ADC (0–2 mm shell-1)	−0.034	0.787	0.073	0.558
Peritumoral ADC (2–5 mm shell-2)	−0.081	0.523	−0.064	0.611
Peritumoral ADC (5–10 mm shell-3)	−0.059	0.640	−0.041	0.742
Normalized ADC (Intratumoral_rADC)	−0.312	0.010	−0.337	0.006
Normalized ADC (Shell-1_rADC, 0–2 mm)	−0.097	0.437	−0.118	0.344
Normalized ADC (Shell-2_rADC, 2–5 mm)	−0.142	0.258	−0.151	0.226
Normalized ADC (Shell-3_rADC, 5–10 mm)	−0.088	0.487	−0.104	0.408
Intratumoral/peritumoral ADC ratio (0–2 mm shell-1)	−0.346	0.004	−0.459	<0.001
Intratumoral/peritumoral ADC ratio (2–5 mm shell-2)	−0.291	0.017	−0.318	0.009
Intratumoral/peritumoral ADC ratio (5–10 mm shell-3)	−0.268	0.028	−0.301	0.013

Spearman’s rank correlation analysis was used to evaluate associations between ADC-based parameters and histological grade as well as Ki-67 proliferation index. Negative correlation coefficients indicate decreasing ADC values or ADC ratios with increasing tumor grade or proliferative activity.

## Data Availability

The MRI datasets generated and analyzed during the current study are not publicly available due to institutional data protection policies but are available from the corresponding author on reasonable request.
